# An Elevated METS-IR Index Is Associated With Higher Asthma Morbidity and Earlier Age of First Asthma in US Adults: Results Based on a Cross-Sectional Study

**DOI:** 10.3389/fendo.2022.920322

**Published:** 2022-07-11

**Authors:** Yan Chen, Junping Yang, Kexing Han, Yan Wang, Cuixia Zhuang, Laxiang Zhu, Mingwei Chen

**Affiliations:** ^1^ Department of General Practice, Wuhu City Second People’s Hospital, Wuhu, China; ^2^ Department of Infectious Diseases, the First Affiliated Hospital of Anhui Medical University, Hefei, China; ^3^ Department of Endocrinology, the First Affiliated Hospital of Anhui Medical University, Hefei, China

**Keywords:** asthma onset age, METS-IR index, cross-sectional study, NHANES, metabolic syndrome, asthma prevalence

## Abstract

**Objective:**

The purpose of this study was to evaluate whether there is a correlation between the METS-IR index and asthma among Americans.

**Methods:**

In an attempt to establish the relationship between the METS-IR index and asthma prevalence and age at first onset of asthma, we conducted a logistic regression analysis, subgroup analysis, and dose-response curve analysis using the National Health and Nutrition Examination Survey (NHANES) database.

**Results:**

In model 3, each unit increase in METS-IR index led to 1.5% increase in asthma prevalence (OR= 1.015, 95% CI: 1.012, 1.018) and an earlier age of onset of asthma by 0.057years (β= -0.057, 95% CI: -0.112, -0.002).Stratified analysis determined that an increase in METS-IR index was associated with asthma prevalence in almost all subgroups, except in the group where it was not known whether a blood relative had asthma, and a positive linear relationship was found between METS-IR index and asthma prevalence, as well as a linear negative relationship with age at asthma onset.

**Conclusion:**

Despite the fact that a direct causal relationship cannot be demonstrated, a higher METS-IR index is positively related to asthma prevalence and correspondingly may result in asthma onset at younger ages.

## Introduction

A significant number of people suffer from asthma, a chronic respiratory condition. Asthma exacerbations can occur even when asthma is well managed, resulting in significant declines in lung function and quality of life for the patient (1). An estimated 334 million people worldwide suffer from asthma according to the Global Burden of Disease Study (GBD) for the period 2008-2010 (2).According to the British National Health Service (NHS), asthma prevalence in the United Kingdom (UK) was 9.6 percent in 2016, and asthma-related hospitalizations were approximately 93000 (3). Hospitalizations for asthma due to poorly managed or severe disease account for a substantial portion of direct medical expenditures associated with the condition (4). Globally, asthma has become one of the most prevalent diseases, contributing to a substantial disease burden (5). Identifying risk factors such as smoking, alcohol consumption, air pollution, and occupational exposure can help to prevent asthma significantly (5–7).

With improved living conditions, the number of people with metabolic syndrome is also increasing. This is due to an increase in the consumption of high-fat and high-sugar diets. The metabolic syndrome (MetS) encompasses various metabolic disorders, including obesity (especially abdominal obesity), fasting, postprandial hyperglycemia, hypertension, and dyslipidemia. In the United States, studies have revealed that 250,000 new cases of asthma are associated with obesity every year (8).Accordingly, this relationship fundamentally changes the distribution of asthmatics in the United States: asthma prevalence is 7.1% among lean adults and 11.1% among obese adults. Women are even more affected by this relationship - the prevalence of asthma is 7.9% in lean women and 14.6% in obese women (9).Insulin resistance (IR), one of the central mechanisms of the metabolic syndrome (10, 11), has been implicated in asthma (12, 13).

Currently, the high insulin normoglycemic clamp (HEC) is the gold standard for determining insulin sensitivity in peripheral tissues (14). Due to this method’s complexity, time-consuming nature, and resource-intensive nature, simpler metrics are often used to gauge insulin resistance. An innovative insulin resistance (IR) metric has been developed, the METS-IR index, which is meant to serve as a simple, reliable, reproducible metric marker for assessing insulin resistance (15, 16). It can be hypothesized that the METS-IR index may have an association with asthma as it has been proposed as a marker of IR. Nonetheless, no previous studies have examined the association between METS-IR index and asthma. Consequently, we conducted this study in order to investigate the relevance of the METS-IR index with respect to the prevalence of asthma and age at first onset of asthma in the United States adult population.

## Materials and Methods

### Study Population

The clinical data analyzed in this study were obtained from the National Health and Nutrition Examination Survey (NHANES) from 2001 to 2018. Every two years, the Centers for Disease Control and Prevention (CDC) carried out a cross-sectional survey of the US population. NHANES study protocol was reviewed and approved by the Institutional Review Board of the National Center for Health Statistics (NCHS), and consent forms were signed by participants. We analyzed nine consecutive two-year survey cycles, including data from the asthma questionnaire. We retained information on participants who explicitly responded that they had asthma, along with their age at which they first developed asthma. In total, 91351 individuals completed the questionnaire. Exclusion criteria were as follows: 1. missing FPG (n=63049); 2. missing TG (n=579); 3. age ≤ 19 (n=6026); 4. missing education level (n=21); 5. missing marital information (n=8); 6. missing activity information (n=`5); 7. missing hypertension information (n=71) 8. missing information about diabetes (n=17); 9. missing information about smoking (n=16); 10. missing information about asthma (n=34); 11. missing information about BMI (n=432); 12. METS-IR outlier values (n=1). Finally a total of 21082 cases were included in this study, including 2883 self-reported asthma history.

### Data Collection and Definition

The METS-IR index is designed to be used as a measurement of exposure. METS-IR= Ln[(2 × fasting glucose) + fasting triglycerides] × body mass index)/[Ln(high-density lipoprotein cholesterol)]. Blood samples were processed to determine fasting glucose and fasting total triglyceride levels in the morning after 8.5 hours of fasting. Triglyceride and fasting blood glucose concentrations were determined enzymatically using an automated biochemical analyzer. Serum triglyceride concentrations were measured using the Roche Modular P and Roche Cobas 6000 chemistry analyzers. To measure asthma, questionnaires were used, including information for asthma, which was the answer to the questions “Have you ever been told that you have asthma?” and “Age when you first had asthma?”. Self-reported asthma status has been demonstrated to be accurate. The participant was considered to have asthma if he indicated “yes” that he had the condition. Both the prevalence of asthma and the age at which the first asthma attack occurred were designed as outcome variables.

The potential confounding factors that may affect the association between the METS-IR index and asthma were identified in multivariate adjusted models. Covariates in our study included gender (male/female), age (years), race, education level, poverty to income ratio (PIR), marital status (married or living with partner/single), alcohol consumption (drinking or not), physical activity (vigorous/moderate/below moderate), whether blood relatives had asthma (yes/no), cholesterol level (mg/dl), body mass index (BMI), smoking status (yes/no), blood relatives had asthma(yes/no), hypertension, diabetes, and dietary intake factors including energy intake, fat intake, sugar intake, and water intake. Except in 2001-2002, for which all participants were eligible for two 24-hour dietary recalls. We used the average consumption of the two recalls to conduct our analysis.

### Statistical Methods

Methodology for handling missing values: In this study the missing values were mainly PIR and diet-related data, which we transferred to categorical variables, and the missing values generated new dummy variables. we converted the variables to categorical variables and assessed the variables in tertiles, using the lowest tertile as the reference group. Details of all measurements incorporating study variables are available on the following web site: www.cdc.gov/nchs/nhanes/.

The sampling weights, stratification, and clustering provided in the NHANES study were applied to all statistical analyses to account for the complex, multistage sampling design used in selecting a representative noninstitutionalized U.S. population and to obtain accurate estimates of statistical significance that would not be overstated. Regarding the selection of weights, the principle of the official guidelines provided by NHANES is to first specify the variable that examines the smallest population and then go on to select the weight corresponding to that variable. In this study our data included MEC examination data, in which fasting triglyceride data were used, and according to the weight selection guidelines recommendations, we chose the subweight corresponding to fasting triglyceride (WTSAF2YR). New sampling weights for the combined survey cycles were constructed by dividing the 2-year weights for each cycle by 9 according to the NHANES analysis guidelines (17).

Variables with continuous characteristics were expressed as means together with their standard deviations, and categorical characteristics were expressed as percentages. In order to determine the variability of clinical characteristics among groups, weighted Chi-square tests (categorical variables) and weighted variance analysis (continuous variables with a normally distributed distribution) or weighted Kruskal-Wallis`s H tests (continuous variables with a skewed distribution) were employed. We used multiple logistic regression models (18) to explore the independent relationship between METS-IR index, METS-IR index triple quantile groups, and asthma in three different models. To exclude the problem of cointegration, we used the cointegration test, when VIF greater than 5 was considered to have cointegration problem. In model 1, no adjustment for covariates was made. Model 2 was adjusted for sex, age, and race. Model 3 was adjusted for sex, age, race, education level, poverty-income ratio, marital status, alcohol intake, physical activity, cholesterol, blood relative with or without asthma, smoking status, hypertension, diabetes, energy intake, sugar intake, water intake. Smoothed curve fitting (penalized spline method) and generalized additive model (GAM) regression were performed to further assess the association between METS-IR index and asthma and age at first onset of asthma. If a nonlinear relationship was identified, inflection points were calculated by a likelihood ratio test. Next, METS-IR was transformed into a dichotomous variable according to the level, and we used the inverse probability weighting method (IPTW) to do sensitivity analysis to further verify the stability of the model. On the basis of validating the model stability, we then conducted multiple regression analyses stratified by gender, age, race, hypertension, diabetes and blood relatives had asthma. Probability values less than 0.05 were considered significant. We performed all analyses using the Empower software www.empowerstats.com; X&Y Solutions Inc., Boston, MA, USA) and survey designs R package.

## Results

The demographic characteristics of the participants included in the study are shown in **Table 1**. METS-IR index was 45.108 ± 14.240 in the asthma group, higher than 42.568 ± 12.190 in the normal group, while the proportion of females was higher in the asthma group, 57.953 with asthma, participants in the asthma group were younger in age compared to the non-asthma group, p < 0.001.

**Table 1:** Baseline characteristics of participants, weighted.

Characteristic	Non asthma formers	Asthma formers	P-value
	N=18199	N=2883	
Age (years)	47.185 ± 16.767	45.181 ± 16.927	<0.00001
BMI (kg/m^2^)	28.651 ± 6.580	30.224 ± 7.835	0.00014
Serum Cholesterol (mg/dl)	194.433 ± 41.369	193.881 ± 44.088	0.51551
METS-IR Index	42.568 ± 12.190	45.108 ± 14.240	<0.00001
Gender (%)			<0.00001
Male	49.244	42.047	
Female	50.756	57.953	
Race (%)			<0.00001
Mexican American	14.080	10.541	
White	67.944	69.123	
Black	10.851	12.836	
Other Race	7.125	7.499	
Education Level (%)			0.22757
Less than high school	20.142	19.952	
High school	27.82	26.464	
More than high school	52.038	53.584	
Marital Status (%)			<0.00001
Cohabitation	65.694	59.137	
Solitude	34.306	40.863	
Alcohol (%)			0.01393
Yes	62.951	65.704	
No	21.235	19.821	
Unclear	15.814	14.475	
High Blood Pressure (%)			<0.00001
Yes	30.637	36.563	
No	69.363	63.437	
Diabetes (%)			0.00064
Yes	8.585	10.49	
No	91.415	89.51	
Smoked			<0.00001
Yes	44.968	51.544	
No	55.032	48.456	
Physical Activity (%)			0.68303
Never	29.095	28.843	
Moderate	31.978	32.772	
Vigorous	38.927	38.385	
Blood Relative Has Asthma (%)			<0.00001
Yes	17.824	42.808	
No	80.212	53.679	
Unclear	1.965	3.513	
Total Kcal (%)			0.06913
Tertile 1	25.646	27.239	
Tertile 2	29.778	27.753	
Tertile 3	32.487	32.27	
Unclear	12.089	12.738	
Total Sugar (%)			0.01264
Tertile 1	25.233	25.086	
Tertile 2	26.327	23.721	
Tertile 3	26.544	28.025	
Unclear	21.896	23.168	
Total Water (%)			0.03568
Tertile 1	25.482	27.576	
Tertile 2	30.625	29.537	
Tertile 3	31.803	30.149	
Unclear	12.089	12.738	
Total Fat (%)			0.09473
Tertile 1	24.748	26.431	
Tertile 2	29.905	29.221	
Tertile 3	33.258	31.611	
Unclear	12.089	12.738	
PIR (%)			<0.00001
<1.3	18.72	24.539	
≥1.3,<3.5	34.778	33.355	
≥3.5	40.186	35.652	
Unclear	6.316	6.454	

Statistically significant: p<0.05;Mean+SD for continuous variables: P value was calculated by weighted linear regression model. %for Categorical variables: P value was calculated by weighted chi-square test. BMI, Body mass index(kg/m^2^); PIR, Ratio of family income to poverty.

### Asthma Prevalence Was Associated With a Higher METS-IR Index

According to the collinearity check results, the VIF value for the variable total fat intake is greater than 5, there is a collinearity problem, and the variable total fat intake is removed in the regression model. It was observed that the METS-IR index and asthma are positively correlated, based upon the results of fully adjusted model 3 (OR= 1.015, 95% CI: 1.012, 1.018).In addition, we converted the METS-IR index from a continuous (continuous) variable to a categorical (categorical) variable for sensitivity analysis. There was a significant 50.2% increase in asthma prevalence in Tertile 3 compared with the lowest METS-IR index (Tertile 1) (OR =1.502,95%CI:1.352, 1.669), respectively), as shown in **Table 2**.

**Table 2:** Analysis between METS-IR index with asthma prevalence.

Characteristic	Model 1 OR (95%CI)	Model 2 OR (95%CI)	Model 3 OR (95%CI)
METS-IR Index	1.018 (1.015, 1.021)	1.019 (1.016, 1.022)	1.015 (1.012, 1.018)
Categories			
Tertile 1	1	1	1
Tertile 2	1.008 (0.910, 1.116)	1.130 (1.018, 1.254)	1.099 (0.986, 1.223)
Tertile 3	1.538 (1.398, 1.691)	1.691 (1.534, 1.865)	1.502 (1.352, 1.669)

Model 1=no covariates were adjusted. Model 2=Model 1+age, gender, race were adjusted. Model3=Model 2+, diabetes, blood pressure, education, marital status, PIR, total water, total kcal, total sugar, smoked, physical activity, alcohol use, serum cholesterol, blood relative has asthma were adjusted.

Next, we divided METS-IR into two categories by levels and then added a new propensity score weighting method to construct an inverse probability weighting analysis (IPTW) to calculate the weights and calculate the effect of the weighted METS-IR index on the prevalence of asthma. The results are as follows, in **Table 1**, the difference between the baseline information of the two groups after IPTW is no longer statistically significant, indicating that the IPTW weighting direction was successfully constructed, and then we used the IPTW method to construct the regression model, and the effect values remained significant (Modle4) (**Supplementary Table 1**, **Table 3**).

**Table 3:** Analysis between METS-IR index with asthma prevalence by using IPTW.

Characteristic	Model 1 OR (95%CI)	Model 2 OR (95%CI)	Model 3 OR (95%CI)	Model 4OR (95%CI)
Lower	1	1	1	1
Higher	1.332 (1.231, 1.441)	1.427 (1.316, 1.547)	1.298 (1.191, 1.416)	1.287(1.181, 1.402)

Model 1=no covariates were adjusted. Model 2=Model 1+age, gender, race were adjusted. Model3=Model 2+, diabetes, blood pressure, education, marital status, PIR, total water, total kcal, total sugar, smoked, physical activity, alcohol use, serum cholesterol, blood relative has asthma were adjusted. Model 4=after IPTW.

### METS-IR’s Dose-Response and Threshold Effects on Asthma Prevalence

**Figure 1:**
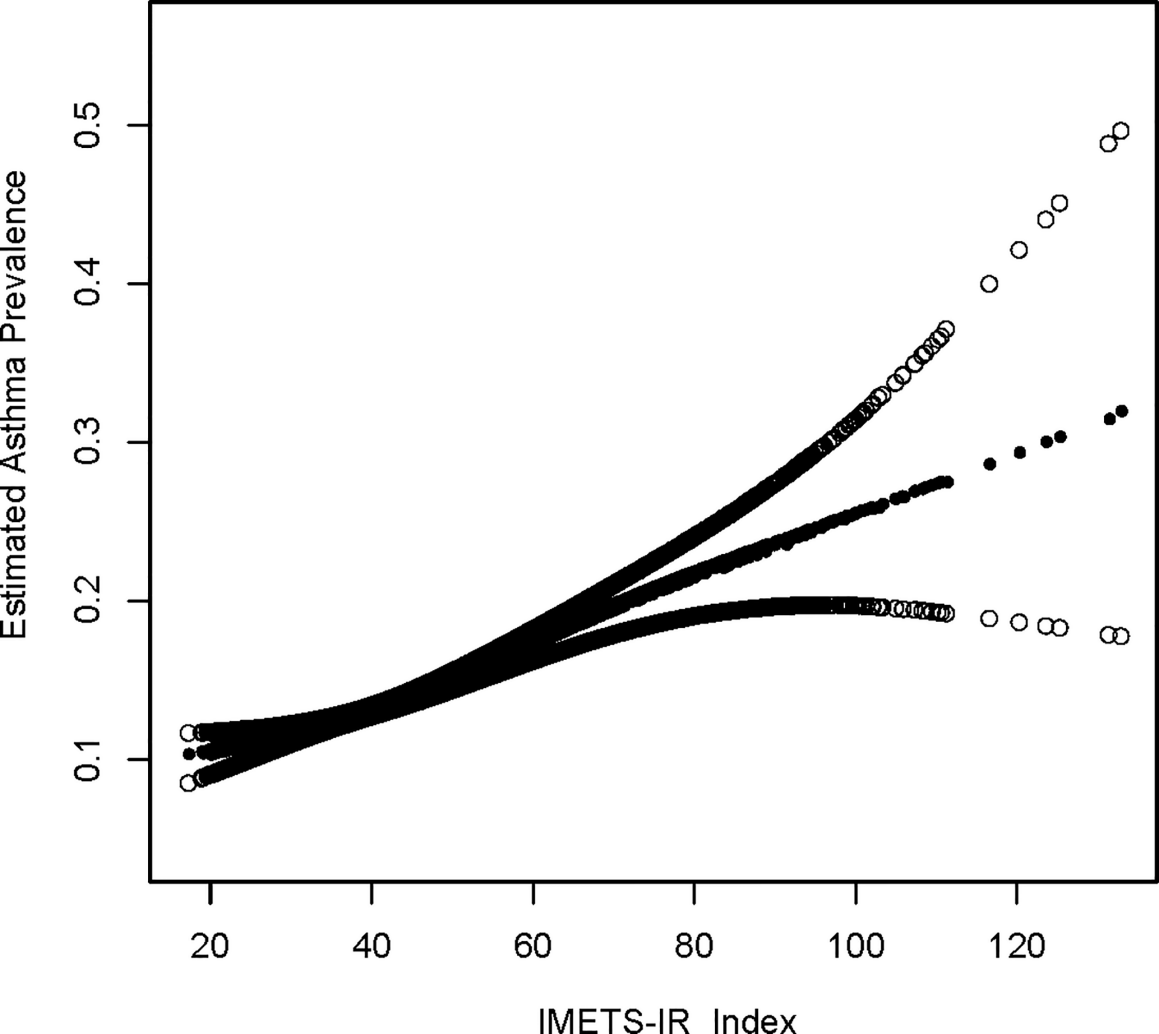
Density dose-response relationship between METS-IR index with asthma prevalence. The area between the upper and lower dashed lines is represented as 95% CI. Each point shows the magnitude of the METS-IR index and is connected to form a continuous line. Adjusted for all covariates except effect modifier.

An additive generalized model and smoothed curve fitting were used to explore the relationship between METS-IR index and asthma. According to our results, there is a positive linear correlation between METS-IR index and asthma (**Figure 1**).

### Subgroup Analysis

Subgroup analyses were conducted in order to examine the robustness of the association between METS-IR and asthma. Based on the results of the subgroup analysis, increased METS-IR was positively associated with asthma prevalence, except in the group where it was unclear whether it was inherited from blood relatives (**Table 4**).

**Table 4:** Subgroup analysis between METS-IR index with asthma prevalence.

Characteristic	Model 1 OR (95%CI)	Model 2 OR (95%CI)	Model 3 OR (95%CI)
Subgroup analysis stratified by gender			
Male	1.010 (1.005, 1.015)	1.012 (1.007, 1.017)	1.010 (1.005, 1.015)
Female	1.024 (1.020, 1.028)	1.024 (1.020, 1.028)	1.019 (1.015, 1.023)
Subgroup analysis stratified by age (years)			
20-39	1.014 (1.010, 1.019)	1.015 (1.010, 1.020)	1.014 (1.008, 1.019)
40-59	1.020 (1.015, 1.026)	1.022 (1.016, 1.027)	1.014 (1.008, 1.020)
≥60	1.023 (1.017, 1.030)	1.025 (1.018, 1.031)	1.022 (1.015, 1.029)
Subgroup analysis stratified by hypertension			
YES	1.018 (1.014, 1.023)	1.016 (1.012, 1.021)	1.015 (1.010, 1.020)
NO	1.015 (1.011, 1.019)	1.017 (1.013, 1.022)	1.016 (1.011, 1.020)
Subgroup analysis stratified by diabetes			
YES	1.023 (1.016, 1.031)	1.021 (1.013, 1.028)	1.017 (1.009, 1.025)
NO	1.016 (1.013, 1.020)	1.017 (1.014, 1.021)	1.015 (1.011, 1.018)
Subgroup analysis stratified by race			
Mexican American	1.016 (1.009, 1.024)	1.017 (1.010, 1.025)	1.016 (1.008, 1.024)
White	1.018 (1.014, 1.023)	1.019 (1.015, 1.024)	1.015 (1.010, 1.020)
Black	1.016 (1.010, 1.022)	1.016 (1.010, 1.022)	1.011 (1.005, 1.018)
Other Race	1.035 (1.025, 1.046)	1.037 (1.026, 1.048)	1.028 (1.016, 1.041)
Subgroup analysis stratified by blood relative has asthma			
Yes	1.017 (1.012, 1.022)	1.018 (1.013, 1.023)	1.016 (1.011, 1.021)
No	1.016 (1.012, 1.020)	1.017 (1.013, 1.022)	1.014 (1.010, 1.018)
Unclear	1.017 (1.001, 1.034)	1.016 (0.999, 1.033)	1.017 (0.998, 1.037)

Model 1=no covariates were adjusted. Model 2=Model 1+age, gender, race were adjusted.

Mode3=adjusted for all covariates except effect modifier means only in model 3.

### METS-IR Index Is Associated With Earlier Age of Asthma Onset

With a fully adjusted model 3, we found that each 1-unit increase in the METS-IR index in the subgroup analysis was associated with an age at asthma onset 0.057 years younger. Only in the non-diabetic group was an increased METS-IR index found to be associated with an earlier age of asthma onset(β= -0.057, 95% CI: -0.112, -0.002) (**Table 5**, **Supplementary Table 2**).

**Table 5:** Analysis between METS-IR index with onset age of asthma.

Characteristic	Model 1 β (95%CI)	Model 2 β (95%CI)	Model 3 β (95%CI)
METS-IR Index	0.101 (0.046, 0.156)	0.098 (0.043, 0.152)	-0.057 (-0.112, -0.002)
Subgroup analysis stratified by diabetes			
YES	-0.012 (-0.149, 0.125)	-0.017 (-0.155, 0.120)	-0.004 (-0.142, 0.134)
NO	0.035 (-0.027, 0.097)	0.032 (-0.030, 0.093)	-0.062 (-0.122, -0.002)

Model 1=no covariates were adjusted. Model 2=Model 1+gender, race were adjusted. Model3=adjusted for all covariates except effect modifier. The subgroup analysis was stratified by diabetes and hypertension, not adjusted for the stratification variable itself

### The Dose Response and Threshold Effects of METS-IR on Onset Age of Asthma

**Figure 2:**
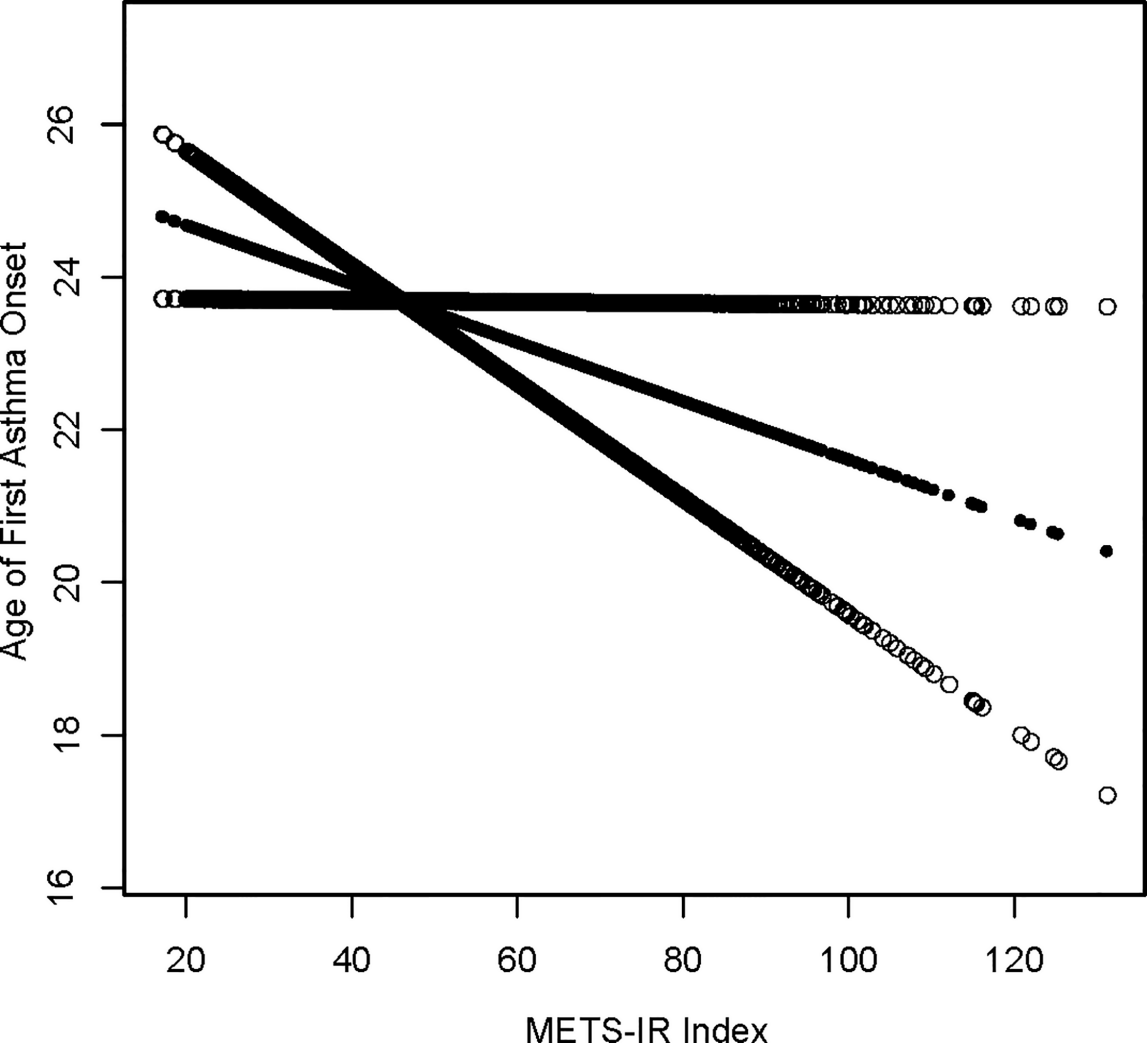
Density dose-response relationship between METS-IR index with onset age of prevalence. The area between the upper and lower dashed lines is represented as 95% CI. Each point shows the magnitude of the METS-IR index and is connected to form a continuous line. Adjusted for all covariates except effect modifier.

A generalized additive model and smoothed curve fitting were used to explore the relationship between the METS-IR index and asthma. In our study, we discovered a negative linear relationship between METS-IR index and onset age of asthma (**Figure 2**).

## Discussion

This is a cross-sectional study using NHANES data to confirm the association between insulin resistance-related indices and asthma prevalence and age at first asthma onset. Our generalized additive model and smoothed curve fit were used to illustrate the relationship between METS-IR and asthma prevalence. According to the results of the METS-IR index and asthma prevalence (**Figure 1**), we discovered a positive linear relationship between the METS-IR index and asthma prevalence.

Researchers are increasingly using observational studies to estimate the effects of treatments, exposures, and interventions on health outcomes. In randomized controlled trials, randomization ensures that, on average, treated subjects do not differ systematically from control subjects in terms of measured and unmeasured baseline characteristics. Thus, treatment effects can be estimated by directly comparing outcomes between treatment groups. However, nonrandomized studies of the effect of treatment on outcomes may be subject to treatment selection bias in that treated subjects differ systematically from controls. Therefore, in non-randomized studies, treatment effects cannot be estimated by simply comparing (19). Baseline information for asthmatic and non-asthmatic investigators remained systematically different after the use of weighted analysis in this study. To eliminate the differences between the two groups(**Supplementary Table 1**), we used the inverse probability weighting method (IPTW) of propensity scoring methods, which has now been shown to be effective in reducing selection bias in observational studies (19, 20). We first turned METS-IR into a dichotomous variable with high or low levels, and after using IPTW, the baseline information between the two was indeed no longer statistically different. The traditional logistic regression was then done using the IPTW method, and the results suggested that the effect values of METS-IR on asthma prevalence in this study remained stable after using IPTW, which in turn suggests that the results of the logistic regression in the original model 3 are valid (**Table 3**).

It is especially important to prevent asthma onset since asthma is a chronic disease that is burdensome to society in terms of morbidity, quality of life, and medical care costs (21).Identifying populations that are most receptive to the METS-IR index can improve asthma prevention. Therefore, we conducted sensitivity subgroup analyses and the results indicated that the positive association between METS-IR index and asthma prevalence was applicable to almost all populations. We showed significant positive correlations in age, gender, race, hypertension, diabetes and presence of blood relatives with asthma subgroups, which also indicates the prevalence of METS-IR index use for asthma population. Furthermore, we observed that increasing age, females, hypertensive, and diabetic populations have higher prevalences, which is in accordance with previous findings (12, 22–25)

According to epidemiological studies, asthma is a heterogeneous disease, and there are several clinical subgroups depending on age of onset, duration of disease, and clinical features (26). Literature indicates that the longer a person suffers from asthma, the more likely they are to decline in respiratory function and their prognosis will be adverse (27). Hence, primary prevention strategies that delay asthma exacerbations are likely to significantly improve the clinical outcome of asthma patients. In this study, it was also demonstrated the importance of METS-IR for age at first asthma attack. We found that each 1-unit increase in METS-IR index was associated with a 0.057-year earlier age of asthma onset. The smoothing curve fitting even indicated a linear negative correlation between METS-IR and the age of first asthma onset. Currently, no study has reported this finding. We hypothesized that treatment and management of IR at an early age might serve to improve or prevent asthma onset. Moreover, we performed subgroup analysis, and elevated METS-IR in non-diabetic populations was associated with an earlier age of asthma onset (**Table 5**).This interesting phenomenon may seem difficult to accept, but similar results have been found in studies in other fields. Iran discovered that non-hypertensive people’s IR causes cardiovascular disease, but that hypertensive people’s IR has no such impact (28). Another Japanese study discovered that raising IR levels in non-diabetic adults increases the risk of coronary heart disease and stroke (29). According to Hedblad et al. (30), the HOMA-IR distribution of non-diabetic adults has the 75th percentile (2.12 for males, 1.80 for women) (for women). In patients with these HOMA-IR scores, the risk of infarction is much greater. These findings imply that in non-diabetic and non-hypertensive groups, IR is more likely to have more severe repercussions. Despite the fact that the research objects are different, this outcome suggests that our findings may be correct to some extent. However, this finding is limited by the small sample size and requires further confirmation by a multicenter prospective study of a large sample.

METS-IR was first mentioned in 2018 and is considered to be a reliable and intuitive predictor of IR (14, 15, 31). In recent years, multiple studies have indicated that insulin resistance can cause asthma onset or exacerbation. Park et al. have reported that metabolic syndrome is linked to asthma onset through IR and inflammation in the elderly (13). Kim et al. have also concluded that IR contributes to bronchial hyperresponsiveness (32), and in children with asthma, insulin resistance is the most significant factor contributing to airway hyperresponsiveness instead of obesity (33). According to Thuesen et al., insulin resistance can influence asthma-like symptoms in adults (12). Moreover, insulin resistance has also been reported to cause systemic inflammation, which may be responsible for severe asthma exacerbations (34). Adenosine triphosphate production may be impaired by IR by reducing glucose utilization and altering lipid metabolism in muscle, resulting in muscle weakness. This may impair airflow, contributing to asthmatic symptoms (35). These studies suggest that elevated IR levels are associated with asthma development, but the fact that the METS-IR index is positively correlated with IR levels may indicate that high METS-IR scores are associated with increased asthmatic risk.

Several advantages can be attributed to our study. A representative sample of the US population is collected in the National Health and Nutrition Examination Survey from 2001- 2018 based on a well-designed study protocol with extensive quality assurance and quality control. As a second step, we controlled for confounding covariates to ensure that our results are reliable and applicable to a broad range of individuals. We acknowledge, however, that the study has certain limitations. First of all, our study was a cross-sectional study using the NHANES database, and a causal relationship between METS-IR index and asthma could not be established. Secondly, a questionnaire was used to evaluate the diagnosis of asthma, which is susceptible to recall bias, while detailed clinical variables such as personal medication histories and asthma type classifications were not recorded and should be further investigated. Despite these limitations, a key strength of this study is that it proposes a new IR index associated with increased asthma prevalence and asthma onset and age at first asthma attack.

## Summary

An increased METS-IR index is associated with an increased prevalence of asthma and an earlier age at which the disease first manifests. Hypothetically, treatment and management of IR at a young age may delay the age of asthma onset and improve or mitigate the onset of asthma, but a causal relationship cannot be clearly established.

## Data Availability Statement

The datasets presented in this study can be found in online repositories. The names of the repository/repositories and accession number(s) can be found in the article/**Supplementary Material**.

## Ethics Statement

The studies involving human participants were reviewed and approved by The NCHS Research Ethics Review Committee approved the NHANES survey protocol (https://www.cdc.gov/nchs/nhanes/irba98.htm). The patients/participants provided their written informed consent to participate in this study.

## Author Contributions

Data analysis and manuscript writing: YC, MC. Study design and statistical advice: YC, JY. Manuscript editing: JY, KH. Validation and review: YW, CZ, LZ. Quality control: LZ, MC. All authors agreed on the journal to which the article was to be submitted and agreed to take responsibility for all aspects of the work.

## Funding

This work was supported by the Natural Science Foundation of Anhui Province(2108085MH269).

## Conflict of Interest

The authors declare that the research was conducted in the absence of any commercial or financial relationships that could be construed as a potential conflict of interest.

## Publisher’s Note

All claims expressed in this article are solely those of the authors and do not necessarily represent those of their affiliated organizations, or those of the publisher, the editors and the reviewers. Any product that may be evaluated in this article, or claim that may be made by its manufacturer, is not guaranteed or endorsed by the publisher.

## Abbreviations

NHANES, National Health and Nutrition Examination Survey; BMI, body mass index; PIR, ratio of family income to poverty; NCHS, National Center for Health Statistics; CI, confidence interval; OR, odds ratio; MetS, metabolic syndrome; IR, insulin resistance; TG, Triglyceride; TC, Cholesterol; FPG, fasting plasma glucose
