# Supplementary material for: An Elevated METS-IR Index Is Associated With Higher Asthma Morbidity and Earlier Age of First Asthma in US Adults: Results Based on a Cross-Sectional Study
Source: Front Endocrinol (Lausanne). 2022 Jul 11;13:920322. doi: 10.3389/fendo.2022.920322 (PMC9309520; doi:10.3389/fendo.2022.920322)
Supplement: Supplementary file 1 [file DataSheet_1.docx]

Supplementary Table1.Comparison of baseline data for METS-IR classification by using IPTW

| **Characteristic** | **Before IPTW** | | | **After IPTW** | | |
| --- | --- | --- | --- | --- | --- | --- |
|  | **Lower** | **Higher** | **P-value** | **Lower** | **Higher** | **P-value** |
| Age(years) | 45.8± 17.6 | 48.3 ± 15.9 | <0.00001 | 49.7 ± 19.0 | 49.5 ± 17 | 0.4 |
| Serum Cholesterol (mg/dl) | 194.7± 40.6 | 197.9 ± 43.5 | <0.00001 | 196.0 ± 43.1 | 195.8± 42.6 | 0.6 |
| Gender(%) |  |  | <0.00001 |  |  | 0.9 |
| Male | 42.7 | 53.7 |  | 48.6 | 48.6 |  |
| Female | 57.3 | 46.3 |  | 51.4 | 51.4 |  |
| Race(%) |  |  | <0.00001 |  |  | 0.9 |
| Mexican American | 11.4 | 16.3 |  | 25.8 | 25.5 |  |
| White | 70.0 | 66.7 |  | 43.6 | 43.8 |  |
| Black | 10.1 | 11.5 |  | 20.9 | 20.9 |  |
| Other Race | 8.5 | 5.5 |  | 9.7 | 9.7 |  |
| Education Level(%) |  |  | <0.00001 |  |  | 0.8 |
| Less than high school | 17.5 | 22.0 |  | 28.8 | 28.5 |  |
| High school | 26.1 | 29.8 |  | 26.9 | 2.7 |  |
| More than high school | 56.4 | 48.2 |  | 44.4 | 44.5 |  |
| Marital Status(%) |  |  | <0.00001 |  |  | 0.9 |
| [Cohabitation](#keyfrom=E2Ctranslation) | 62.2 | 66.9 |  | 60.8 | 60.7 |  |
| [Solitude](#keyfrom=E2Ctranslation) | 37.8 | 33.1 |  | 39.2 | 39.3 |  |
| Alcohol(%) |  |  | <0.00001 |  |  | 0.9 |
| Yes | 64.8 | 60.3 |  | 58.3 | 58.4 |  |
| No | 19.0 | 22.5 |  | 24.8 | 24.6 |  |
| Unclear | 16.2 | 17.3 |  | 16.9 | 1.7 |  |
| High Blood Pressure(%) |  |  | <0.00001 |  |  | 0.6 |
| Yes | 21.7 | 40.3 |  | 35.1 | 34.8 |  |
| No | 78.3 | 59.7 |  | 64.9 | 65.2 |  |
| Diabetes(%) |  |  | <0.00001 |  |  | 0.3 |
| Yes | 3.7 | 14.5 |  | 12.5 | 12.1 |  |
| No | 96.3 | 85.5 |  | 87.5 | 87.9 |  |
| Smoked |  |  | <0.00001 |  |  | 0.9 |
| Yes | 25.1 | 33.2 |  | 45.6 | 45.6 |  |
| No | 30.9 | 32.5 |  | 54.4 | 54.4 |  |
| Physical Activity(%) | 44.0 | 34.3 |  |  |  | 0.9 |
| Never |  |  | <0.00001 | 36.1 | 35.9 |  |
| Moderate | 44.3 | 47.1 |  | 30.6 | 30.7 |  |
| Vigorous | 55.7 | 52.9 |  | 33.3 | 33.4 |  |
| Blood Relative Has Asthma(%) |  |  | 0.0 |  |  | 0.8 |
| Yes | 21.0 | 21.9 |  | 20.9 | 21.0 |  |
| No | 79.0 | 78.1 |  | 79.1 | 79.0 |  |
| Fast Time(%) |  |  | <0.00001 |  |  | 0.9 |
| Yes | 61.9 | 61.0 |  | 61.6 | 61.4 |  |
| No | 27.2 | 25.4 |  | 27.4 | 27.5 |  |
| Unclear | 10.9 | 13.6 |  | 11.0 | 11.1 |  |
| PIR(%) |  |  | <0.00001 |  |  | 0.9 |
| ＜1.3 | 18.0 | 20.9 |  | 28.3 | 28.2 |  |
| ≥1.3,＜3.5 | 32.0 | 34.9 |  | 35.2 | 35.2 |  |
| ≥3.5 | 43.1 | 37.6 |  | 28.4 | 28.4 |  |
| Unclear | 6.8 | 6.6 |  | 8.1 | 8.2 |  |
| Total Kcal(%) |  |  | <0.00001 |  |  | 0.9 |
| Tertile 1 | 26.3 | 24.8 |  | 28.5 | 28.3 |  |
| Tertile 2 | 29.2 | 29.0 |  | 28.2 | 28.2 |  |
| Tertile 3 | 30.4 | 33.2 |  | 27.8 | 28.1 |  |
| Unclear | 14.2 | 12.9 |  | 15.5 | 15.4 |  |
| Total Sugar(%) |  |  | 0.0 |  |  | 0.9 |
| Tertile 1 | 25.1 | 25.1 |  | 25.4 | 25.4 |  |
| Tertile 2 | 25.8 | 25.3 |  | 25.3 | 25.3 |  |
| Tertile 3 | 25.5 | 26.7 |  | 25.2 | 25.3 |  |
| Unclear | 23.6 | 22.9 |  | 24.1 | 23.9 |  |
| Total Water(%) |  |  | <0.00001 |  |  | 0.9 |
| Tertile 1 | 26.7 | 23.9 |  | 28.4 | 28.2 |  |
| Tertile 2 | 29.9 | 30.0 |  | 28.1 | 28.2 |  |
| Tertile 3 | 29.2 | 33.2 |  | 28.0 | 28.2 |  |
| Unclear | 14.2 | 12.9 |  | 15.5 | 15.4 |  |
| Total Fat(%) |  |  | <0.00001 |  |  | 0.9 |
| Tertile 1 | 26.1 | 23.3 |  | 28.3 | 28.3 |  |
| Tertile 2 | 29.9 | 28.7 |  | 28.2 | 28.1 |  |
| Tertile 3 | 29.8 | 35.1 |  | 28.0 | 28.2 |  |
| Unclear | 14.2 | 12.9 |  | 15.5 | 15.4 |  |

Supplementary Table 2 Subgroup analysis between METS-IR index with onset age of asthma

| **Characteristic** | **Model** 1 β(95%CI) | **Model** 2 β(95%CI) | **Model** 3 β(95%CI) |
| --- | --- | --- | --- |
| Subgroup analysis stratified by gender | | | |
| Male | 0.145 (0.054, 0.236) | 0.137 (0.046, 0.228) | 0.008 (-0.083, 0.098) |
| Female | 0.066 (-0.002, 0.135) | 0.074 (0.005, 0.142) | -0.048 (-0.117, 0.021) |
| Subgroup analysis stratified by race | | | |
| Mexican American | 0.140 (-0.001, 0.282) | 0.136 (-0.005, 0.278) | 0.019 (-0.122, 0.159) |
| White | 0.096 (0.018, 0.174) | 0.101 (0.023, 0.178) | -0.035 (-0.114, 0.043) |
| Black | 0.103 (-0.005, 0.212) | 0.075 (-0.034, 0.185) | -0.050 (-0.161, 0.061) |
| Other Race | 0.068 (-0.117, 0.253) | 0.072 (-0.112, 0.257) | -0.129 (-0.317, 0.058) |
| Subgroup analysis stratified by blood relative has asthma | | | |
| Yes | 0.116 (0.065, 0.167) | 0.119 (0.068, 0.170) | 0.031 (-0.021, 0.084) |
| No | 0.101 (0.047, 0.154) | 0.098 (0.045, 0.151) | -0.022 (-0.075, 0.031) |
| Subgroup analysis stratified by hypertension | | | |
| YES | -0.009 (-0.070, 0.052) | -0.015 (-0.076, 0.046) | -0.056 (-0.118, 0.006) |
| NO | 0.026 (-0.021, 0.073) | 0.024 (-0.023, 0.071) | -0.039 (-0.085, 0.008) |

Model 1=no covariates were adjusted.

Model 2=Model 1+gender,race were adjusted.

Model3=Model 2+,diabetes,blood pressure,education,marital status,,,total water,total kcal,total fat,total sugar,smoked,physical activity,alcohol use,serum cholesterol were adjusted.

The subgroup analysis was not adjusted for the stratification variable itself
